# Checkpoint Inhibitor-Associated Diabetes Mellitus Following Rapid Pancreatic Metastasis Resolution: Implications for Endocrine Toxicities in Immunotherapy

**DOI:** 10.1016/j.aed.2025.08.014

**Published:** 2025-09-02

**Authors:** Sinead Cadogan, Sadhbh Doherty, Deirdre Kelly, Nigel Glynn

**Affiliations:** 1 Department of Diabetes and Endocrinology, Mater Misericordiae University Hospital, Dublin, Ireland; 2 Department of Radiology, Mater Misericordiae University Hospital, Dublin, Ireland; 3 Department of Oncology, Mater Misericordiae University Hospital, Dublin, Ireland

**Keywords:** anti-PDL1, diabetes mellitus, diabetic ketoacidosis, drug-related side effects and adverse reactions, immune checkpoint inhibitors

## Abstract

**Background/Objective:**

Checkpoint inhibitor-associated autoimmune diabetes mellitus (CIADM) is a rare complication of immunotherapy, often leading to abrupt insulin deficiency and diabetic ketoacidosis. We present a case arising during atezolizumab therapy following rapid pancreatic metastasis resolution, suggesting a link between tumor regression, local immune activation, and beta-cell injury.

**Case Report:**

A 67-year-old woman was diagnosed with extensive-stage small-cell lung cancer and a pancreatic metastasis. She began treatment, including atezolizumab, a programmed death-ligand 1 inhibitor. Imaging after 7 weeks of treatment showed complete resolution of the pancreatic lesion. At week 35, she presented with polyuria, polydipsia, and vomiting. Labs confirmed diabetic ketoacidosis, with marked hyperglycemia, low C-peptide, and negative diabetes-related autoantibodies. She was diagnosed with CIADM and required prolonged intravenous insulin followed by basal-bolus therapy. Computed tomography imaging 2 months later demonstrated a 52% reduction in pancreatic volume.

**Discussion:**

This case highlights the fulminant onset of autoimmune diabetes during immunotherapy, with profound insulin deficiency and pancreatic atrophy. Its onset following rapid pancreatic tumor resolution suggests that localized immune activation may have contributed to beta-cell destruction.

**Conclusion:**

CIADM may emerge as an immune-related adverse event in patients with rapid pancreatic tumor response. Early recognition is essential, particularly in those with pronounced tumor regression, to ensure timely diagnosis and management.

**Highlights**

• Checkpoint inhibitor-associated autoimmune diabetes mellitus (CIADM) is a rare but serious complication of immune checkpoint inhibitor therapy
• CIADM presents with rapid beta-cell loss, leading to abrupt insulin deficiency and frequent diabetic ketoacidosis
• Pancreatic atrophy may reflect severe immune injury in CIADM
• Rapid pancreatic tumor response may trigger CIADM via local immune activation mechanisms
• Early diagnosis and intensive insulin therapy are critical for management

**Clinical Relevance**

Checkpoint inhibitor-associated autoimmune diabetes mellitus is a rare but serious endocrine toxicity. This case highlights a potential mechanistic link between rapid pancreatic tumor regression and beta-cell destruction, and underscores the need for early recognition and glucose monitoring in patients receiving immunotherapy, especially as use of checkpoint inhibitors continues to expand.

## Introduction

Immune checkpoint inhibitors (ICIs) have revolutionized cancer treatment by enhancing antitumor immune responses. However, their use can result in immune-related adverse events (irAEs), including endocrine toxicities. Among these, checkpoint inhibitor-associated autoimmune diabetes mellitus (CIADM) is a rare but serious complication, often presenting with diabetic ketoacidosis (DKA) and requiring lifelong insulin therapy.^1^ CIADM occurs in approximately 1% of ICI-treated patients.^2^ Diagnosis is based on abrupt hyperglycemia with low or undetectable C-peptide levels, indicating profound insulin deficiency. Unlike classic type 1 diabetes (T1DM), CIADM frequently occurs in the absence of diabetes-related autoantibodies and without a prodromal phase. The underlying mechanism is thought to involve programmed death-ligand 1 (PD-L1) inhibition disrupting peripheral immune tolerance, triggering T cell–mediated beta-cell destruction.

CIADM has been increasingly reported in lung cancer, predominantly in non–small cell lung cancer (SCLC). Reports in SCLC are scarce. In the IMpower133 trial of atezolizumab plus chemotherapy for extensive-stage SCLC, only one case of T1DM was reported among 201 participants—an incidence of 0.5%^3^—highlighting its rarity in this setting.

We describe a case of autoimmune diabetes mellitus following atezolizumab in a patient with extensive-stage SCLC and a pancreatic metastasis. Diabetes developed shortly after rapid resolution of the pancreatic lesion, suggesting a possible link between local immune activation and beta-cell injury.

## Case Report

**Fig. 1:**
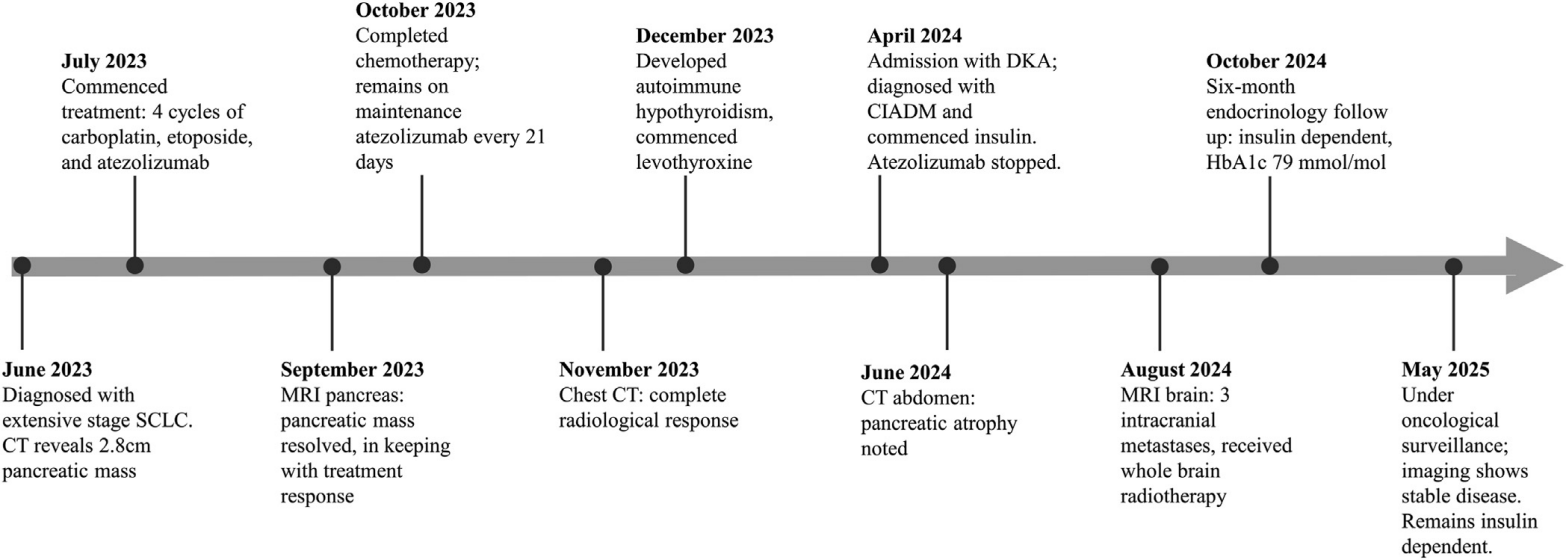
Clinical timeline of oncologic treatment, imaging, and endocrine events.

A 67-year-old woman was diagnosed with extensive-stage SCLC. Initial computed tomography (CT) imaging showed a right hilar mass, mediastinal lymphadenopathy, pleural effusion, and a 2.4 × 2.2 cm enhancing mass within the body of the pancreas, concerning for metastasis. Histology of the hilar mass confirmed SCLC. She had a history of chronic obstructive pulmonary disease, and a body mass index of 27 kg/m^2^. There was no history of diabetes; baseline glycosylated hemoglobin (HbA1c) was 41 mmol/mol (reference range: 20–41 mmol/mol). Family history was notable for a brother with type 2 diabetes and several relatives (mother, sister, daughter) with hypothyroidism. A summary of key events is shown in Figure 1.

She received 4 cycles of carboplatin, etoposide, and atezolizumab; dexamethasone was given on day 1 of cycles 2–4 as an antiemetic. Maintenance atezolizumab (1200 mg every 3 weeks) followed. Serum glucose monitoring during treatment visits showed consistently normal values. By week 7, pancreatic magnetic resonance imaging showed complete lesion resolution. By 14 weeks, contrast-enhanced CT of the chest similarly confirmed complete thoracic disease remission. At 5 months into treatment, she developed autoimmune hypothyroidism due to ICI-induced thyroiditis and was started on levothyroxine (Table 1).

**Table 1:** Thyroid Function Tests by Week of Atezolizumab Therapy

Investigations	Reference range	Week 0	Week 14	Week 22	Week 25
Free thyroxine (pmol/l)	9.0 – 20.0	13.8	16.9	8.7 ↓	<5.4 ↓
Free triiodothyronine (pmol/l)	2.6 – 6.0	4.1	5.1	3.2	<1.9 ↓
Thyroid-stimulating hormone (mIU/l)	0.35 – 4.94	2.92	0.15 ↓	8.36 ↑	96.14 ↑
Thyroid peroxidase antibody (IU/ml)	<5.6				711 ↑

At week 35 (following 11 doses of atezolizumab), 28 weeks after radiological resolution of the pancreatic metastasis, she reported 4 days of polyuria, polydipsia, abdominal pain, and vomiting. She was tachycardic (heart rate 107 bpm) and clinically dehydrated. Labs confirmed DKA (Table 2). Despite marked hyperglycemia, HbA1c was only modestly elevated, consistent with abrupt-onset diabetes. C-peptide was profoundly low, and diabetes autoantibodies were negative, supporting a diagnosis of CIADM. CT imaging on admission showed no evidence of pancreatitis.

**Table 2:** Biochemical and Hormonal Investigations at Presentation with Diabetic Ketoacidosis

Investigations	Result	Reference range
Venous pH	7.14 ↓	7.35 – 7.45
pCO2 (kPa)	4.62 ↓	6.0 – 8.0
HCO3 (mmol/l)	11.8 ↓	22.4 – 25.8
Anion gap (mmol/l)	21.4 ↑	6 – 16
Glucose (mmol/l)	25.4 ↑	3.7 – 6.0
Ketone (mmol/l)	7.7 ↑	<0.6
Sodium (mmol/l)	131 ↓	133 – 146
Potassium (mmol/l)	4.7	3.3 – 5.0
Chloride (mmol/l)	102	95 – 108
Urea (mmol/l)	10.2 ↑	2.8 – 8.6
Creatinine (μmol/l)	81	46 – 86
eGFR (mL/min/1.73m^2^)	69	>60
HbA1c (mmol/mol)	50 ↑	20 – 42
C-peptide (nmol/l)	0.03 ↓	0.26 – 1.73
GAD antibody (IU/ml)	<5	0 – 9
Islet cell antibody	Negative	Negative
Amylase (IU/l)	21 ↓	28 – 97
Morning cortisol (nmol/l)	336	150 – 450

Abbreviations: *eGFR* = estimated glomerular filtration rate; *GAD* = glutamic acid decarboxylase; *HbA1c* = glycosylated hemoglobin.

She was treated with intravenous insulin, resolving the ketoacidosis, but experienced recurrent DKA despite subcutaneous bridging. Prolonged IV insulin was needed before transitioning to a basal-bolus regimen (48 units/day; 0.8 units/kg/d). She was discharged with multidisciplinary follow-up. Atezolizumab was discontinued, with the patient opting for surveillance.

**Fig. 2:**
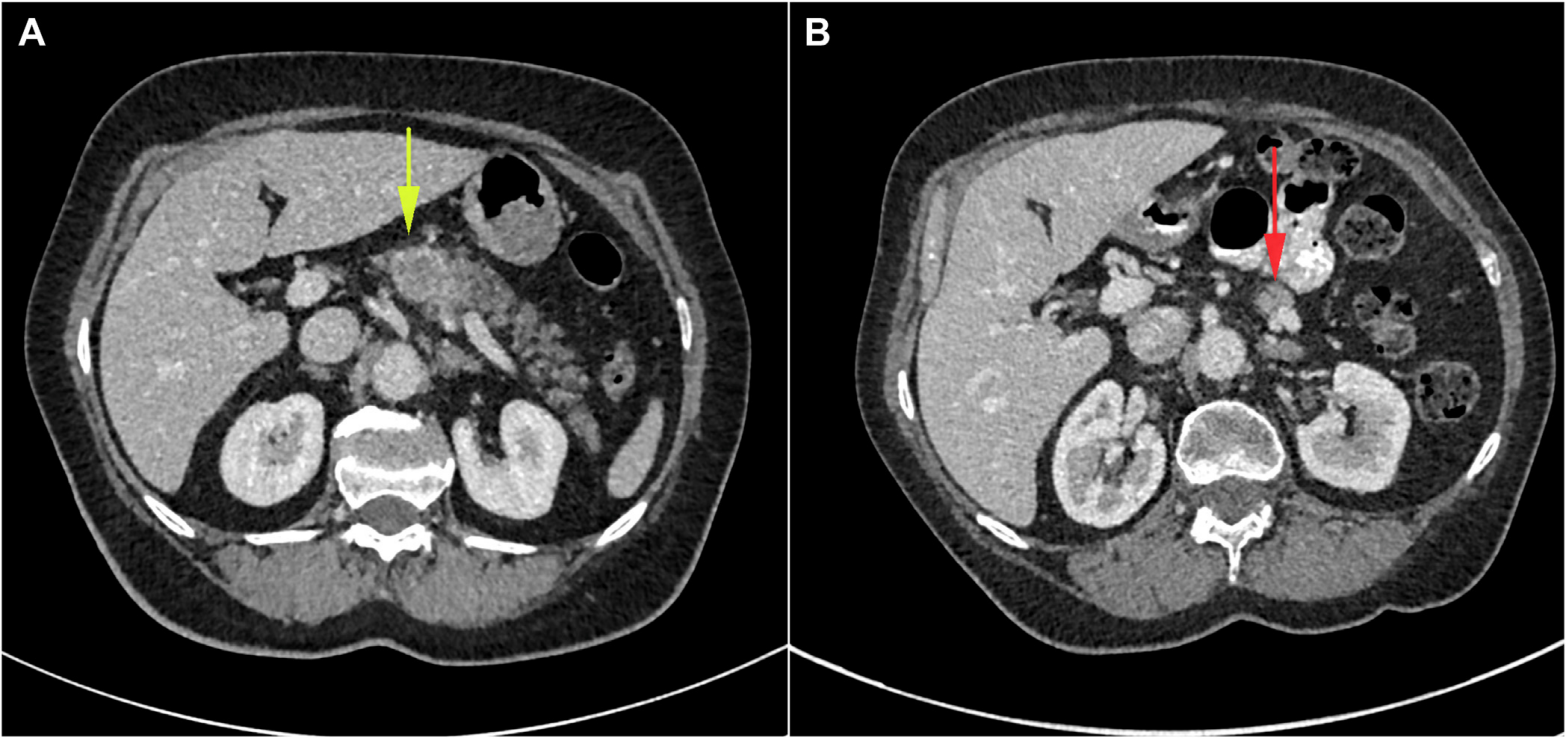
Contrast-enhanced computed tomography (CT) images of the pancreas before and after treatment with immune checkpoint inhibitor. (A) Yellow arrow: Pancreatic metastasis in the body of the pancreas measuring 2.4 cm in maximum axial diameter (baseline image). (B) Red arrow: Pancreatic atrophy and resolution of the metastasis (post-treatment image).

Contrast-enhanced CT 2 months later demonstrated a diffuse 52% reduction in pancreatic volume, decreasing from 71.4 mL at baseline to 37.1 mL (Fig. 2). Volumetric assessment was performed using the TeraRecon navigation browser, with pancreatic borders manually delineated in the axial plane via the freehand region-growing tool. At 6-month follow-up, she remained insulin-dependent with ongoing glycemic instability (HbA1c was 79 mmol/mol), requiring continued multidisciplinary input. Repeat C-peptide testing 1 month after diagnosis was undetectable (<0.01 nmol/L). No evidence of pancreatic exocrine insufficiency was noted. Thirteen months after her initial cancer diagnosis, she developed brain metastases treated with whole-brain radiotherapy. Two years post–SCLC diagnosis, she is alive with stable oncologic disease and continues endocrine and oncology follow-up.

## Discussion

This case describes CIADM induced by atezolizumab in a patient with SCLC and a rapidly resolving pancreatic metastasis. Unlike classic T1DM, which often presents gradually and with a partial remission phase, CIADM involves near-complete beta-cell destruction at onset, evidenced by low or undetectable C-peptide levels in >90% of cases and a markedly higher DKA rate (69.7% vs 6%).^4^ The absence of a ‘honeymoon’ phase and early glycemic instability, as seen here, highlight the importance of early endocrinology input and intensive insulin therapy. CIADM can resemble fulminant T1DM, a subtype more common in East Asian populations.^5^ Our patient met all 3 Japanese diagnostic criteria for fulminant T1DM, suggesting shared features such as abrupt beta-cell loss.^5^ Although less frequently described in non-Asian individuals, ICI therapy may induce a similar immune mechanism independent of genetic predisposition.

CIADM is rarely reported in SCLC, potentially due to lower PD-L1 expression and a more immunosuppressive tumor microenvironment when compared to non–SCLC.^6^

Autoantibodies are present in only 40.4% of CIADM cases, compared with 90% in T1DM.^4^ This likely reflects T cell–mediated beta-cell destruction, bypassing traditional B cell–driven autoimmunity. Thus, C-peptide decline and new-onset hyperglycemia may be more reliable indicators than antibodies.^4^ Emerging biomarkers such as cytokine profiles, T-cell analysis, and HLA typing show promise for risk stratification.^7^, ^8^, ^9^ For example, human leukoctye antigen DR4—a T1DM-susceptibility allele—was present in 76% of one CIADM cohort.^10^ These tools could guide early metabolic monitoring and personalized immunotherapy. IA-2 and ZnT8 antibodies, though not assessed here, appear to have low yield when GAD is negative. In a review of 42 CIADM cases, only 3 patients had IA-2 or ZnT8 antibodies—all GAD-positive—while no cases showed isolated IA-2 or ZnT8 positivity.^11^

The rapid resolution of pancreatic metastasis within 7 weeks suggests a robust local immune response, possibly involving cytotoxic T-cell activity against both tumor and surrounding normal pancreatic tissue. This phenomenon aligns with the inflammatory tumor microenvironment theory, which proposes that immune responses within tumor-infiltrated tissues can expose or amplify local antigens, promoting bystander activation and collateral tissue injury.^12^ In the pancreas, tumor lysis and inflammation may upregulate PD-L1 expression on islet cells—a protective mechanism intended to suppress autoreactive T-cells.^13^^,^^14^ Experimental models have shown that PD-L1 expression protects against T1DM and that its inhibition facilitates islet infiltration by CD8^+^ T-cells, leading to beta-cell loss.^15^

These observations support a “two-hit” model: (1) local inflammation primes immune activity via PD-L1 expression and T-cell recruitment, ^16^^,^^17^ and (2) PD-L1 inhibition releases cytotoxic T-cells, triggering β-cell autoimmunity. In this case, the lysing metastasis may have served as the first hit, with subsequent checkpoint inhibition providing the second hit, enabling a targeted autoimmune β-cell attack, leading to abrupt CIADM onset.

Pancreatic volume declined by 52% postdiabetes onset. CIADM has been associated with greater atrophy than ICI-treated controls (41% vs 6%).^18^ The more substantial reduction observed in our patient suggests particularly severe immune-mediated injury. Pancreatic atrophy is also observed in new-onset T1DM, with one study reporting a 26% volume reduction,^19^ but the loss appears more pronounced in CIADM. Importantly, our patient had a normal baseline volume (71.4 mL), excluding pre-existing atrophy.^20^

The development of autoimmune hypothyroidism illustrates the frequent co-occurrence of endocrine irAEs; nearly half of CIADM cases involve another endocrinopathy, most commonly thyroid dysfunction.^21^ Multisystem irAEs may correlate with improved cancer outcomes, possibly reflecting a stronger immune response.^21^

Although our patient opted for surveillance and discontinued immunotherapy, guidelines allow reinitiation if glycemic control stabilizes.^2^ In this case, cessation after 13 cycles was based on disease stability and patient preference, underscoring the importance of individualized care.

In summary, this case underscores the fulminant presentation of CIADM, necessitating intensive insulin therapy and close multidisciplinary management. The temporal association between rapid tumor regression and β-cell failure points to localized immune activation as a possible trigger. Significant pancreatic atrophy further supports severe, targeted injury. Early tumor response should prompt clinical vigilance, with timely oncology–endocrinology collaboration critical for recognition and management of this rare irAE. Future research should investigate the interplay between pancreatic lesions, volume loss, and immune activation to improve understanding of CIADM pathogenesis and identify patients at risk.

## Patient Consent

Written informed consent for publication of their clinical details was obtained from the patient.

## Disclosure

The authors have no conflicts of interest to disclose.

## References

[1] Kotwal A., Kennedy R., Kikani N., Thosani S., Goldner W., Shariff A. (2024). Endocrinopathies associated with immune checkpoint inhibitor use. Endocr Pract.

[2] Brahmer J.R., Abu-Sbeih H., Ascierto P.A. (2021). Society for immunotherapy of cancer (SITC) clinical practice guideline on immune checkpoint inhibitor-related adverse events. J Immunother Cancer.

[3] Horn L., Mansfield A.S., Szczęsna A. (2018). First-line atezolizumab plus chemotherapy in extensive-stage small-cell lung cancer. New Engl J Med.

[4] Wu L., Tsang V., Menzies A.M. (2023). Risk factors and characteristics of checkpoint inhibitor–associated autoimmune diabetes mellitus (CIADM): a systematic review and delineation from type 1 diabetes. Diabetes Care.

[5] Imagawa A., Hanafusa T., Awata T. (2012). Report of the committee of the Japan diabetes society on the research of fulminant and acute-onset type 1 diabetes mellitus: new diagnostic criteria of fulminant type 1 diabetes mellitus (2012). J Diabetes Invest.

[6] Lee Y.S., Lim J.H., Ryu W. (2021). The clinical impact of three validated PD-L1 immunohistochemistry assays as a prognostic factor in small cell lung cancer. Transl Lung Cancer Res.

[7] Inaba H., Kaido Y., Ito S. (2022). Human leukocyte antigens and biomarkers in type 1 diabetes mellitus induced by immune-checkpoint inhibitors. Endocrinol Metab.

[8] Shalit A., Sarantis P., Koustas E., Trifylli E.M., Matthaios D., Karamouzis M.V. (2023). Predictive biomarkers for immune-related endocrinopathies following immune checkpoint inhibitors treatment. Cancers.

[9] Ostmeyer J., Park J.Y., von Itzstein M.S. (2023). T-cell tolerant fraction as a predictor of immune-related adverse events. J Immunother Cancer.

[10] Stamatouli A.M., Quandt Z., Perdigoto A.L. (2018). Collateral damage: insulin-dependent diabetes induced with checkpoint inhibitors. Diabetes.

[11] Clotman K., Janssens K., Specenier P., Weets I., De Block C.E.M. (2018). Programmed cell Death-1 inhibitor–induced type 1 diabetes mellitus. J Clin Endocrinol Metab.

[12] Lv B., Wang Y., Ma D. (2022). Immunotherapy: reshape the tumor immune microenvironment. Front Immunol.

[13] Ansari M.J.I., Salama A.D., Chitnis T. (2003). The programmed death-1 (PD-1) pathway regulates autoimmune diabetes in nonobese diabetic (NOD) mice. J Exp Med.

[14] Osum K.C., Burrack A.L., Martinov T. (2018). Interferon-gamma drives programmed death-ligand 1 expression on islet β cells to limit T cell function during autoimmune diabetes. Sci Rep.

[15] Mourad D., Azar N.S., Eid A.A., Azar S.T. (2021). Immune checkpoint inhibitor-induced diabetes mellitus: potential role of T cells in the underlying mechanism. Int J Mol Sci.

[16] Quandt Z., Young A., Anderson M. (2020). Immune checkpoint inhibitor diabetes mellitus: a novel form of autoimmune diabetes. Clin Exp Immunol.

[17] Cho Y.K., Jung C.H. (2023). Immune-checkpoint inhibitors-induced type 1 diabetes mellitus: from its molecular mechanisms to clinical practice. Diabetes Metab J.

[18] Wu L., Carlino M.S., Brown D.A. (2024). Checkpoint inhibitor-associated autoimmune diabetes mellitus is characterized by C-peptide loss and pancreatic atrophy. J Clin Endocrinol Metab.

[19] Williams A.J.K., Thrower S.L., Sequeiros I.M. (2012). Pancreatic volume is reduced in adult patients with recently diagnosed type 1 diabetes. J Clin Endocrinol Metab.

[20] Djuric-Stefanovic A., Masulovic D., Kostic J., Randjic K., Saranovic D. (2012). CT volumetry of normal pancreas: correlation with the pancreatic diameters measurable by the cross-sectional imaging, and relationship with the gender, age, and body constitution. Surg Radiol Anat.

[21] Zhang J., Gao A., Wang S. (2024). Correlation between immune-related adverse events and efficacy of PD-(L)1 inhibitors in small cell lung cancer: a multi-center retrospective study. Respir Res.

